# Endoplasmic Reticulum Stress Signalling Induces Casein Kinase 1-Dependent Formation of Cytosolic TDP-43 Inclusions in Motor Neuron-Like Cells

**DOI:** 10.1007/s11064-019-02832-2

**Published:** 2019-07-06

**Authors:** David A. Hicks, Laura L. Cross, Ritchie Williamson, Marcus Rattray

**Affiliations:** 1 School of Pharmacy and Medical Sciences, Faculty of Life Sciences, University of Bradford, Richmond Road, Bradford, BD7 1DP UK; 2 Present Address: Division of Neuroscience & Experimental Psychology, Faculty of Biology, Medicine and Health, School of Biological Sciences, University of Manchester, AV Hill Building, Oxford Road, Manchester, M13 9PT UK

**Keywords:** Motor neuron disease, TDP-43, CK1

## Abstract

Motor neuron disease (MND) is a progressive neurodegenerative disease with no effective treatment. One of the principal pathological hallmarks is the deposition of TAR DNA binding protein 43 (TDP-43) in cytoplasmic inclusions. TDP-43 aggregation occurs in both familial and sporadic MND; however, the mechanism of endogenous TDP-43 aggregation in disease is incompletely understood. This study focused on the induction of cytoplasmic accumulation of endogenous TDP-43 in the motor neuronal cell line NSC-34. The endoplasmic reticulum (ER) stressor tunicamycin induced casein kinase 1 (CK1)-dependent cytoplasmic accumulation of endogenous TDP-43 in differentiated NSC-34 cells, as seen by immunocytochemistry. Immunoblotting showed that induction of ER stress had no effect on abundance of TDP-43 or phosphorylated TDP-43 in the NP-40/RIPA soluble fraction. However, there were significant increases in abundance of TDP-43 and phosphorylated TDP-43 in the NP-40/RIPA-insoluble, urea-soluble fraction, including high molecular weight species. In all cases, these increases were lowered by CK1 inhibition. Thus ER stress signalling, as induced by tunicamycin, causes CK1-dependent phosphorylation of TDP-43 and its consequent cytosolic accumulation.

**Electronic supplementary material:**

The online version of this article (10.1007/s11064-019-02832-2) contains supplementary material, which is available to authorized users.

## Introduction

Motor neuron disease (MND, also known as amyotrophic lateral sclerosis) is a progressive, incurable and ultimately fatal neurodegenerative disease [1]. Ongoing loss of motor neurons in the brainstem, motor cortex and spinal cord leads to muscle weakness, then subsequently paralysis and death [2]. In a minority of cases (approximately 10%), there is a familial background, with mutations in the genes encoding proteins such as superoxide dismutase 1 (SOD1) [3], TAR DNA binding protein 43 (TDP-43) [4], fused in sarcoma (FUS) [5, 6], and C9ORF72 [7, 8]. However, the vast majority of cases are sporadic, with no discovered genetic basis [1].

One of the key pathological hallmarks of MND (both sporadic and familial) is the presence of TDP-43 inclusions in motor neurons [9, 10], although not in SOD1-linked MND [10] In familial MND with mutations in the *TARDBP* gene (encoding TDP-43), the disease-causing mutations cluster in the TDP-43 C-terminal region [4]. TDP-43 aggregation, which can be caused by mutations in a minority of cases, has been demonstrated to interfere with nucleocytoplasmic transport and compounds capable of blocking TDP-43 aggregation in *C. elegans* have been discovered which improve viability [11–16]. It is not yet clear whether the TDP-43 aggregates result in toxicity via loss of function of the nuclear protein, or whether the aggregates confer a toxic gain of function [17–19].

The mechanisms underlying TDP-43 aggregation in sporadic MND are incompletely characterized, although phosphorylation of TDP-43 is likely to be important [20]. TDP-43 is a substrate for a number of protein kinases—casein kinase 1 (CK1) has been identified as a direct TDP-43 kinase, including at S409/410 [21–25], and its overexpression can induce TDP-43 phosphorylation [26]. Furthermore, custom synthesized CK1δ inhibitors were able to reduce TDP-43 phosphorylation and protect cultured cells against ethacrynic acid challenge, which induces neuroblastoma cell death by mediating the phosphorylation of TDP-43 [27]. In addition, there has been substantial interest in developing CK1 inhibitors for MND [28–30].

While the specific pathological drivers of sporadic MND are largely uncharacterized, TDP-43 inclusions are somewhat reminiscent of stress granules (SGs) [31]—many cellular stresses have been shown to cause formation of SGs, which stain positive for T-intracellular antigen-1 cytotoxic granule-associated RNA binding protein-like 1 (TIAR) and Ras GTPase-activating protein-binding protein 1 (G3BP1) [32, 33]. SGs are seen in MND spinal cord and G3BP1-positive inclusions are found in the brains of mice expressing the G_4_C_2_ hexanucleotide expansion found in the *C9ORF72* non-coding region, which also exhibit TDP-43 deposition [34, 35]. However, it is not clear whether TDP-43 inclusions are SG subtypes or whether they are functionally distinct [32, 36, 37]. Optogenetic induction of SGs can potentiate TDP-43 accumulation [38], but TDP-43 can also function as a regulator of stress granule dynamics [39], indicative of a bidirectional relationship.

ER stress and subsequent downstream signalling have been strongly linked to MND [40–42]. The ER stress-induced protein CHOP is upregulated [40, 42] as are other markers associated with ER stress: BiP [43], phospho-eIF2α [44], and protein disulfide isomerase (PDI) [45]. In a screen for putative protein biomarkers of MND, the chaperones ERp57, calreticulin, and PDI featured prominently [46], with genetic variants in the latter being reported as risk factors for MND [47]. Morphologically, ER abnormalities in the disease state have also been observed by electron microscopy [43, 48]. A subset of vulnerable motor neurons was also found to be particularly susceptible to ER stress and the subsequent stress-induced death was ameliorated by salubrinal, a drug targeting ER stress [49]. In vitro, tunicamycin is widely used to induce endoplasmic reticulum (ER) stress as it blocks N-glycosylation of proteins [50], which ultimately leads to accumulation of proteins in the ER.

In this study, we show that the ER-stressor tunicamycin induces the accumulation of endogenous TDP-43 into cytoplasmic inclusions. When tunicamycin treatment is administered to NSC-34 cells alongside a CK1 inhibitor, TDP-43 cytoplasmic accumulation is markedly reduced, in addition to its phosphorylation and segregation into the RIPA-insoluble/urea-soluble fraction, which suggests that CK1 plays a role in the mislocalisation of TDP-43 observed under conditions of ER stress.

## Materials and Methods

All chemicals were purchased from Fisher Scientific (Loughborough, Leicestershire, UK) unless otherwise stated. Pharmacological inhibitors were sourced as indicated: tunicamycin (Cat T7765), PF670462 (Cat SML0795) (Sigma-Aldrich, Gillingham, Dorset, UK), sodium arsenite (Cat #12897692), sorbitol (Cat #10396733) (Fisher Scientific), and D 4476 (Cat CAY13305) (Cambridge Bioscience, Cambridge, UK). Dimethyl sulphoxide was used as a vehicle in cell culture treatments.

Primary antibodies used were for the TDP-43 N-terminus (Proteintech Group, Manchester, UK; RRID:AB_615042, 1:500 (ICC) or 1:1000 (IB)), Phospho(409/410)-TDP-43 (Proteintech Group; RRID:AB_66318, 1:500), BiP/Grp78, (Proteintech; RRID: AB_2119855, 1:500), TIAR (BD Biosciences; RRID:AB_397742, 1:500), G3BP1 (Proteintech Group; RRID: AB_2232034, 1:500) and β-actin (Sigma-Aldrich; RRID:AB_476692, 1:1000).

### Cell Culture

Motor neuron-like NSC-34 cells (RRID:CVCL_D356, a kind gift from Dr. Adrian Higginbottom, Sheffield Institute of Translational Neuroscience, Sheffield, UK) [51] were cultured in Dulbecco’s Modified Eagle’s Medium (DMEM Cat BE12-914F, Lonza, Slough, Berkshire, UK), supplemented with 10% foetal bovine serum (FBS) (Cat FCS-SA/500, Labtech, Uckfield, East Sussex, UK), 2 mM l-glutamine (Cat #35050061), 100 U/mL penicillin, and 100 µg/mL streptomycin (combined Cat #15140122) (all Thermo Fisher Scientific, Paisley, Renfrewshire, UK) to a maximum of passage 30. Cells were incubated at 37 °C and 5% CO_2_ in a humidified atmosphere. For differentiation, cells were seeded and cultured in full growth medium (above) for 24 h, then differentiated for 7 days in DMEM-F12 (Cat BE04-687F, Lonza) supplemented with 1% FBS, 100 U/mL penicillin, 100 µg/mL streptomycin, 1% non-essential amino acids and 1 µM retinoic acid (Cat R2625) (Sigma-Aldrich) [52, 53]. For immunofluorescence experiments, cells were cultured on gelatin-coated glass coverslips (Cat G1393, BioReagent, Sigma-Aldrich). Cells were tested for mycoplasma contamination by PCR. NSC-34 cells are not listed on the ICLAC Register of Misidentified Cell Lines.

### Cell Lysis

Separation of the RIPA-soluble and urea-soluble fractions was performed as previously described [54]. In brief, treated cells were washed twice in ice-cold phosphate-buffered saline (PBS) and harvested in PBS. Cells were pelleted at 3000×*g* for 5 min (4 °C) and re-suspended in 6 × volume of lysis buffer (RIPA buffer: 50 mM Tris–HCl (pH 8.0), 150 mM sodium chloride, 1% Igepal CA-630 (NP-40 substitute, Sigma-Aldrich), 0.5% sodium deoxycholate, 0.1% SDS, 1 mM sodium fluoride, 1 mM sodium orthovanadate, and Complete Protease Inhibitor cocktail (Cat #11836170001, Roche Diagnostics, Burgess Hill, West Sussex, UK)). Lysis was performed for 30 min on ice, followed by centrifugation at 100,000×*g* for 30 min (4 °C) to yield the RIPA-soluble fraction as the supernatant. The resultant insoluble pellet was washed in RIPA buffer, re-centrifuged and resuspended in urea-containing buffer (30 mM Tris–HCl (pH 8.5), 7 M urea, 2 M thiourea, 4% CHAPS, 1 mM sodium fluoride, 1 mM sodium orthovanadate, and Complete Protease Inhibitor cocktail) and sonicated, followed by centrifugation at 100,000×*g* for 30 min. The supernatant was taken as the (RIPA) insoluble fraction.

### Determination of Protein Concentration

Protein concentration in the RIPA-soluble fraction was determined using the bicinchoninic acid (BCA) method [55], using a Pierce BCA Protein Assay Kit (Cat #23225, Thermo Fisher Scientific). In brief, protein samples were incubated for 30 min at 37 °C with BCA solution (BCA with 2% (v/v) copper (II) sulphate). Post-incubation, absorbance at 562-nm was measured using a plate reader (ELx800, BioTek, Swindon, UK, UK). Sample concentration was determined using bovine serum albumin (BSA) as a standard at concentrations from 0 to 1 mg/mL. As the BCA assay is incompatible with 7 M urea, the Pierce 660 nm Protein Assay Kit (Cat #22662, Thermo Fisher Scientific;) was used according to the manufacturer’s instructions. In brief, samples were incubated with reagent for 10 min at room temperature before reading the absorbance at 660-nm. The protein concentration was proportional to the absorbance and was calculated relative to a BSA standard curve (0–2 mg/mL).

### SDS-PAGE and Immunoblotting

Protein samples were separated by electrophoresis (120 V for 90 min) on a polyacrylamide gel containing 12% acrylamide in the absence of reducing agent. After SDS-PAGE, proteins were transferred to polyvinylidene fluoride (PVDF) membranes for 75 min at 125 V (Bio-Rad). The PVDF membranes were incubated for 2 h in blocking solution (5% (w/v) milk powder, 2% (w/v) BSA in TBS + 0.1% (v/v) Tween-20 (TBST)) and then incubated overnight in primary antibody (5% (w/v) milk powder (or BSA for phosphorylated epitopes), in TBS). The PVDF membranes were washed 4 × 10 min with TBST before the addition of secondary antibody (HRP-conjugated anti-IgG (Cat #65-6120 and # A16072; 5% (w/v) milk powder in TBST, 1:5000 (Thermo Fisher Scientific)) for 1 h, followed by 4 × 10 min washes with TBST. Protein bands were visualized by chemiluminescence (Clarity Western ECL Blotting Substrate, Cat #1705061, Bio-Rad) using a G:BOX and GeneTools software (Syngene, Cambridge, UK).

### Lambda (λ) Phosphatase Treatment

NSC-34 cells were lysed in minimal lysis buffer (50 mM Tris (pH7.4), 1% Triton X-100 and 0.5% sodium deoxycholate, followed by incubation with λ phosphatase (Cat #P0753L, New England Biolabs, Hitchin, UK) according to the manufacturer’s instructions followed by SDS-PAGE as described.

### Immunofluorescence Microscopy

Cells were cultured on gelatin coated glass coverslips (BioReagent, Sigma Aldrich). After treatment, cells were fixed in 4% paraformaldehyde for 10 min then washed in PBS. Cells were subsequently permeabilised in 0.5% Triton X-100, washed in PBS and incubated with blocking buffer (0.5% fish skin gelatin (Cat G7041, FSG, Sigma) in PBS) for 1 h. Coverslips were incubated with primary antibody for 1 h (in 0.5% FSG, 0.5% Triton X-100 in PBS), washed in PBS and incubated for 1 h in secondary antibody (0.5% FSG, 0.5% Triton X-100 in PBS and either Alexa Fluor 488 or 594 (Cat A-21202 or R37119, Thermo Fisher Scientific). After washing, coverslips were incubated with 1 µg/mL DAPI (Cat #62248, Thermo Fisher) for 5 min and mounted on microscope slides with Prolong Diamond mounting medium (Cat P36961, Thermo Fisher Scientific). Images were collected on a Zeiss Axioimager.M2 upright microscope using 20 × plan apochromat or 63x plan apochromat objectives as indicated and captured using a Coolsnap HQ2 camera (Photometrics, Tucson, AZ, USA) through Micromanager software v1.4.23. Specific band pass filter sets for DAPI, FITC and Texas Red were used to prevent bleed through from one channel to the next. Images were then processed and analysed using ImageJ (NIH, USA).

### Cell Viability Assays

For cell viability assays, NSC-34 cells were cultured in 96-well plates. An aliquot of culture medium was extracted and added to an equivalent volume of lactate dehydrogenase reaction buffer (Pierce LDH Cytotoxicity Assay, Cat # 88953, Thermo Fisher), incubated for 30 min and absorbance read at 490 nm using a plate reader. The cells and remaining medium were incubated with CellTiter 96 AQueous One or Cell Titer Glo (Cat G3580 or G9241, Promega, Southampton, UK) and, respectively, absorbance at 490 nm or luminescence recorded.

### Statistical Analysis

All experiments are n = 3 unless otherwise indicated, where n indicates a biological replicate. Statistical tests were either Student’s *t* test (control versus tunicamycin) or one-way ANOVA (multiple groups) with Tukey’s post hoc test; *p* < 0.05 (*), *p* < 0.01 (**) *p* < 0.001 (***) or *p* < 0.0001 (****). All statistical analyses were performed using GraphPad Prism 7 (GraphPad Software, Inc., La Jolla, CA, USA). D’Agostino Pearson normality testing was performed which showed data (Fig. 2) to be non-normal. However, ANOVA has been shown to be robust even with non-Gaussian distributions [56]. Tests for outliers and sample calculation were not performed.

### Ethical Approval

Institutional ethical approval was not required for this study.

## Results

### Cellular Stress Induces Formation of TDP-43 Inclusions and Stress Granules

**Fig. 1:**
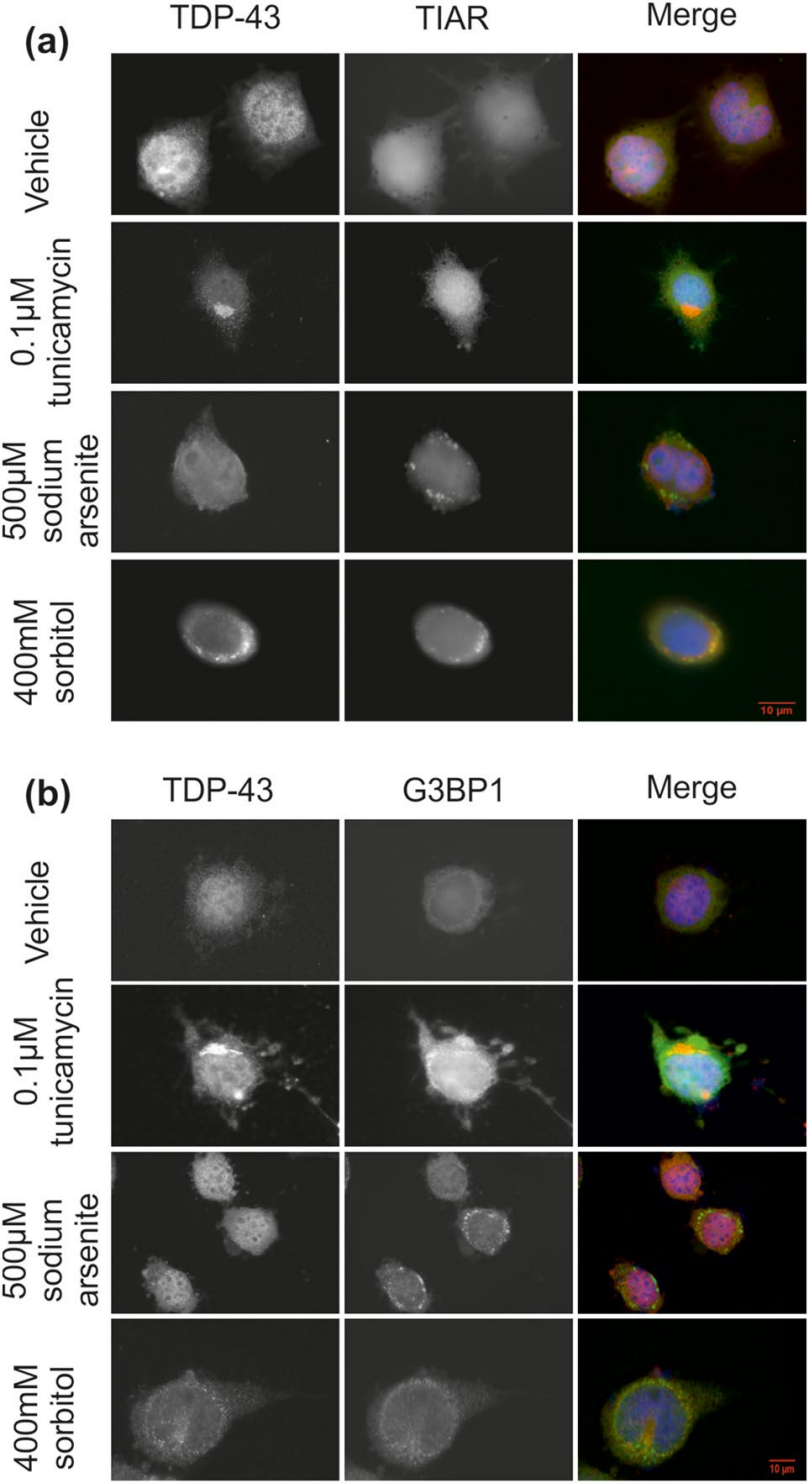
Induction of ER, oxidative and osmotic stress in NSC-34 cells. NSC-34 cells were cultured as described and differentiated for seven days and treated with either vehicle, 0.1 µM tunicamycin (24 h) sodium arsenite (500 µM, 1 h) or 400 mM sorbitol (1 h). Cells were subsequently fixed and immunocytochemistry was performed as described, using primary antibodies against TDP-43, and **a** TIAR or **b** G3BP1 as indicated using a 63 × objective. Scale bars, 10 µm. n = 3 (biological replicates)

Compounds were tested in NSC-34 cells to assess their ability to drive the formation of TDP-43-positive inclusions and stress granules (Fig. 1). The ER stressor, tunicamycin (0.1 µM, 24 h), was able to generate perinuclear TDP-43 inclusions without nuclear depletion, though these were TIAR- and G3BP1- negative (Fig. 1a and b). The tunicamycin-induced TDP-43 inclusions are large in size, often with a single large inclusion per cell (Fig. 1), and resemble the rounded TDP-43 inclusions found in the motor cortex of people with MND [57].

Treatment with sodium arsenite (500 µM, 1 h) resulted in generation of TIAR- and also G3BP1-positive SGs. There were multiple granules per cell, producing an exclusively cytosolic speckled pattern, with a broadly even distribution throughout the cytosol. However, treatment with sodium arsenite failed to generate TDP-43-positive inclusions (Fig. 1). The hyperosmolar stressor, sorbitol (400 mM, 1 h) similarly generated TIAR- and also G3BP1-positive granules, and resulted in TDP-43-positive inclusions (Fig. 1). These inclusions were punctate and cytosolic, visualised as multiple small speckles, and showed a similar morphology to the circumferential TDP-43 inclusions reported by Tan et al. [57], although the inclusions in that study were more skein-like than the multiple punctate structures seen in our work. In addition, Tan et al. used human post-mortem tissue as opposed to the cultured cells use here [57]. Immunocytochemistry showed that only a proportion of the sorbitol-induced SGs also stained positive for TDP-43, indicating at least two subpopulations of inclusions (Fig. 1). Taken together, these data show that cellular stress can result in the accumulation of TDP-43 in cytosolic inclusions, the morphology of which is dependent on the inciting stressor.

### Tunicamycin-Induced ER Stress Does Not Affect TDP-43 Levels or Cell Viability

Tunicamycin induced the most robust formation of TDP-43-positive inclusions and was thus selected for further characterisation. Under conditions of ER stress, chaperones such as Grp78 are known to become activated, a response which is proposed to support correct folding of accumulating proteins [58]. The abundance of the ER chaperone Grp78 (aka BiP) was determined by immunoblotting (Fig. S1). Tunicamycin treatment resulted in a 3.5-fold increase in Grp78 immunoreactivity (Fig. S1a and b), demonstrating that ER stress is occurring in these cells. There were no concomitant increases in abundance of soluble TDP-43 (Fig. S2a and b) and this concentration of tunicamycin had no effect on cell viability, as determined by the CellTiter Glo assay (Fig. S1c). These data show that the selected concentration of tunicamycin induces a robust ER stress response, but does not affect overall cell viability.

### CK1 Inhibition Reduces the Formation of TDP-43 Positive Inclusions

**Fig. 2:**
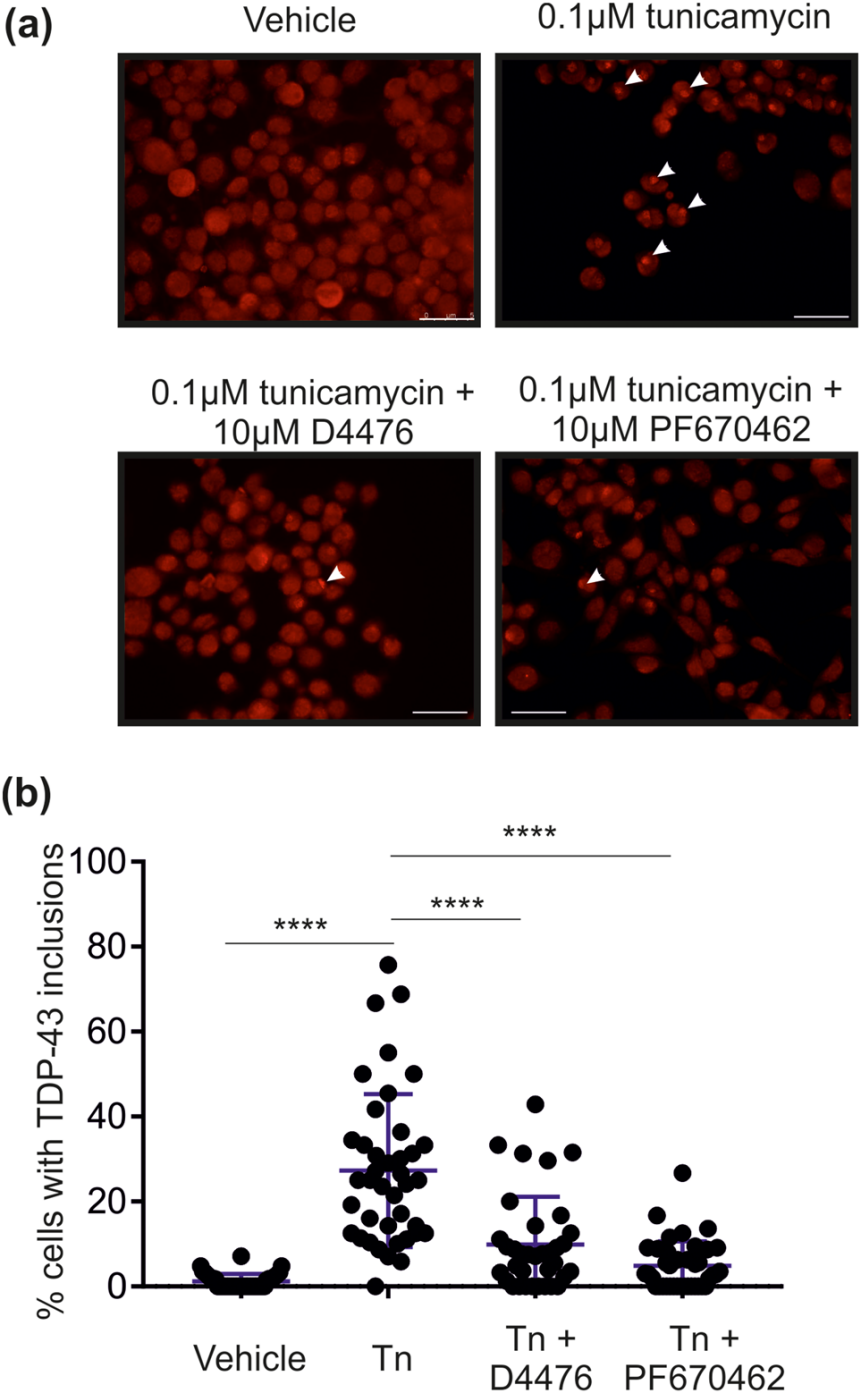
Ability of CK1 inhibitors to block TDP-43 aggregation. NSC-34 cells were treated with 0.1 µM tunicamycin (Tn) ± 10 µM D4476 or 10 µM PF670462, as indicated. Cells were subsequently fixed and **a** immunocytochemistry was performed as described, using primary antibodies against TDP-43 using a 20 × objective. Scale bars, 50 µm and **b** the percentage of cells with cytoplasmic inclusions was calculated. Each data point refers to one field of view, where n = 4 (biological replicates). Statistical significance was assessed using one way ANOVA with Tukey’s post hoc test. Horizontal lines (blue) indicate mean and SD

CK1ε has been identified as the only TDP- 43 kinase able to significantly potentiate the toxic effects of TDP-43 Q331 K over-expression in *Drosophila* [22]. However, CK1δ has also been linked to MND [26]. Therefore, in this study, a two pharmacological inhibitors of CK1 were used, D4476 and PF670462 (both 10 µM, 24 h) [59, 60]. In untreated cells, TDP-43 positive inclusions were extremely sparse (in 1.3% of cells), whereas tunicamycin treatment induced formation of TDP-43 positive inclusions in 27% of cells. Co-incubation of tunicamycin with the CK1 inhibitors significantly reduced the effect of tunicamycin by 64% (D4476) or 82% (PF670462) as determined by percentage cells with TDP-43 positive inclusions (Fig. 2). Incubation of cells with either inhibitor alone had no effect on TDP-43 localisation or inclusion formation (data not shown). These data show that tunicmycin-induced ER stress potentiates TDP-43 accumulation through a CK1-dependent mechanism.

**Fig. 3:**
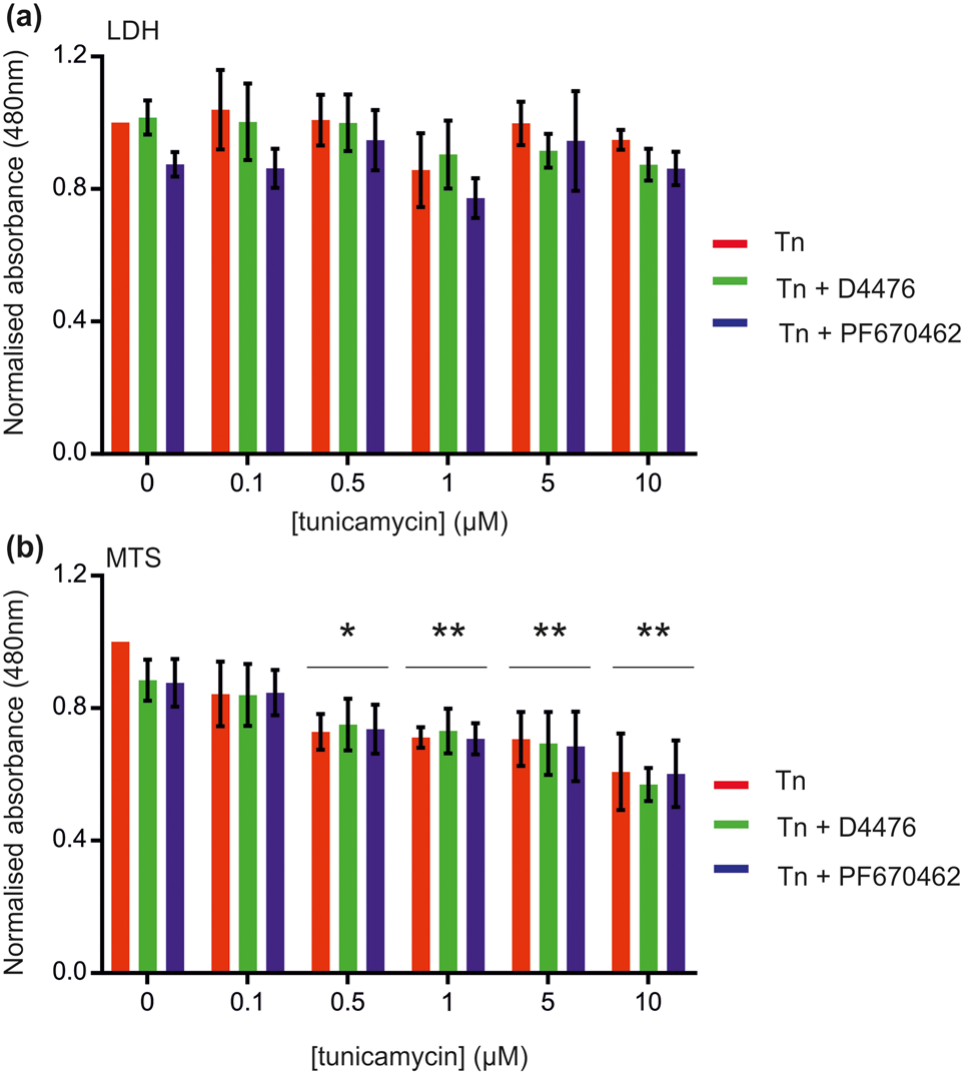
Impact of ER stress on cell viability. Cell viability was assessed using **a** the LDH Cytotoxicity Assay and **b** the CellTiter 96 AQueous One Solution Cell Proliferation Assay. Horizontal lines indicate statistical significance for all groups. Each point represents the mean (n = 3 biological replicates) with error bars as SD. Statistical significance was assessed using one way ANOVA with Tukey’s post hoc test. Significance denotes statistical difference from vehicle (i.e. 0 µM tunicamycin)

In order to test the effects of the CK1 inhibitors on cell viability, a two complementary assays were performed. The LDH cytotoxicity assay was used, which is a measure of extracellular LDH and hence membrane integrity. Tunicamycin treatment had no effect on LDH release, at concentrations up to 10 µM (Fig. 3a). Similarly, co-incubation with the CK1 inhibitors had no effect on LDH release (Fig. 3a). The CellTiter 96 AQueous One Solution Cell Proliferation Assay was used to measure the cells’ ability to reduce the MTS tetrazolium dye to a coloured formazan product. No change in absorbance was seen with treatment of cells with 0.1 µM tunicamycin. However, treatment of cells with tunicamycin at concentrations ranging from 0.5 µM to 10 µM resulted in a significant decrease in absorbance, indicating impaired cellular metabolic capabilities (Fig. 3b). Co-incubation of tunicamycin with either of the CK1 inhibitors failed to rescue the deficiencies in cellular metabolism (Fig. 3b). Thus treatment of NSC-34 cells with concentrations of tunicamycin up to 10 µM does not cause frank cell death. However, at concentrations above 0.5 µM, there is evidence of more subtle metabolic disturbances, which are not reversed by CK1 inhibition.

### CK1 Inhibition Reduces Tunicamycin-Induced TDP-43 Phosphorylation and Accumulation in the RIPA-Insoluble Fraction

Immunocytochemistry indicated that signalling downstream of ER stress induction by tunicamycin resulted in cytoplasmic accumulation of TDP-43. To further characterise this process, cell fractionation (RIPA soluble and RIPA insoluble/urea soluble fractions) and immunoblotting were used as previously reported [54].

**Fig. 4:**
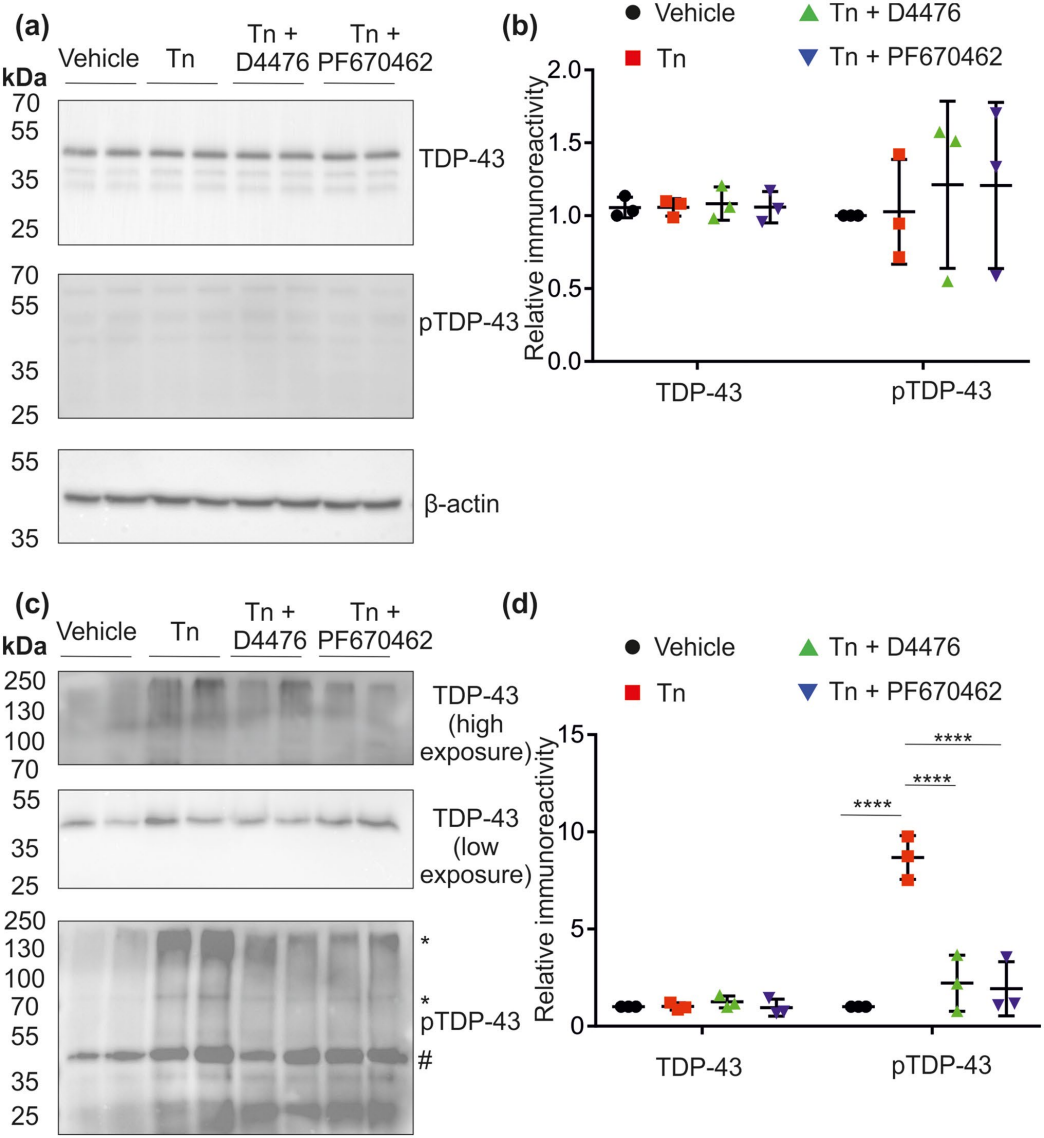
Fractionation and immunoblot analysis of TDP-43. NSC-34 cells were treated with vehicle control or 0.1 µM tunicamycin (Tn) ± 10 µM D4476 or PF670462. **a** RIPA-soluble and **c** RIPA-insoluble/urea-soluble fractions were prepared, followed by SDS-PAGE and immunoblot, probing for TDP-43 and pTDP-43. Densitometric analysis was performed on blots from the soluble fraction (**b**) and the insoluble fraction (**d**). Phospho-TDP-43 densitometry represents the 80 kDa immunoreactive band. * = high molecular weight pTDP-43 and # = monomeric pTDP-43. Each point represents a biological replicate (n = 3) with error bars as SD. Statistical significance was assessed using one way ANOVA with Tukey’s post hoc test. Horizontal lines (black) indicate mean and SD

Immunoblot analysis of the RIPA-soluble fraction showed no significant changes in either TDP-43 or phospho-TDP-43 (S409/S410) (pTDP-43) immunoreactivity with tunicamycin treatment ± CK1 inhibitors (Fig. 4a and b). All total TDP-43 was at a molecular weight consistent with a monomer and this was recapitulated with pTDP-43 immunoblotting, although the latter is demonstrably expressed at very low levels in the soluble fraction. Lysates from this fraction were treated with λ phosphatase and the consequent molecular weight shift in the pTDP-43 monomer confirms its identity, suggesting other bands to be non-specific (Fig. S2a).

In the RIPA-insoluble, urea-soluble fraction, probing for total TDP-43, the predominant immunoreactive band also corresponds to the molecular weight of the monomer. There is no significant change in immunoreactivity with tunicamycin treatment with or without CK1 inhibitors (Fig. 4c and d). In this fraction, immunoblotting for phospho-TDP-43 detected several immunoreactive bands, corresponding to the predicted TDP-43 monomer and TDP-43 dimer, and also higher molecular weight assemblies. The immunoreactive pTDP-43 bands broadly correspond to the pattern of bands detected by total TDP-43 albeit with varying intensities of the different molecular weight species detected. In order to verify the identity of pTDP-43 immunoreactive bands in the RIPA-insoluble fraction, the reducing agent DTT was used (1 mM in sample buffer) on the basis that higher molecular weight TDP-43 species have been suggested to be dependent on thiol linkages [54]. Both the high molecular weight species of pTDP-43 (approximately 250 kDa) and the putative pTDP-43 dimer showed substantial reductions in immunoreactivity in the presence of DTT. No other bands were seen to be sensitive to DTT (Fig. S2b).

Tunicamycin treatment induced significant increases in higher molecular weight pTDP-43 species (Fig. 4c and d). This effect of tunicamycin was reduced by co-incubation with either of the CK1 inhibitors D4476 or PF670462 (Fig. 4c and d). Incubation of cells with either inhibitor alone had no effect on TDP-43 immunoreactivity in the RIPA-soluble or insoluble fractions (data not shown).

Overall, ER stress signalling induced by tunicamycin has no effect on TDP-43 abundance in the RIPA-soluble fraction. However, significant increases in high molecular weight, phosphorylated TDP-43 species are seen in the RIPA-insoluble/urea-soluble fraction after tunicamycin treatment. The abundance of these species is substantially reduced by co-treatment of tunicamycin with a CK1 inhibitor.

## Discussion

Here we show that stress treatments of cultured NSC-34 cells induced different patterns of TDP-43 and TIAR/G3BP1-positive inclusions, supporting the hypothesis that in sporadic MND, TDP-43 aggregation is potentiated by cellular stress. Here we have focussed on chemical induction of ER stress, showing that tunicamycin-induced ER stress signalling can cause profound accumulation of TDP-43 in NSC-34 cells. In both this study and the work by Leggett et al. [61], tunicamycin induces the formation of a small number of large cytoplasmic inclusions, rather than the numerous puncta seen with some other stressors [23]. ER stress has been strongly implicated in the pathogenesis of MND, and our study is among the first to show that ER stress can induce cytoplasmic accumulation of endogenous TDP-43, a major pathological hallmark of MND, although this has been shown using thapsigargin, which also induces ER stress [62, 63] This process is partially blocked by pharmacological inhibition of CK1, suggesting a key role for CK1.

Despite its prominence in MND, ER stress has received modest attention. Our data show that CK1 plays a vital role in TDP-43 cytoplasmic inclusion formation under conditions of chronic ER stress. A role for CK1 is supported in previously published reports [22, 26, 27], which has relevance for the pharmacological targeting of TDP-43 aggregation [14, 27, 28, 30].

The data presented here also offer insight into the relationship between SGs and TDP-43 inclusions. SGs are considered to be protective against acute stress [32]. In our study we find no evidence of co-localisation between the SG markers TIAR and G3BP1 and endogenous TDP-43 following ER stress. This is in line with recent findings showing a lack of association between SG markers and mature TDP-43 inclusions [35]. We also show that stressors can induce formation of TDP-43 inclusions with substantially different morphology, here with osmotic stress giving rise to a different pattern than that induced by ER stress. Our findings support existing evidence that different cellular stressors give rise to differing responses in terms of formation of SGs and TDP-43 inclusions, with a considerable diversity in SGs [64]. This is in agreement with previous work demonstrating the heterogeneity in response to cell stress with regard to TDP-43 inclusion and SG formation [63, 65] TDP-43 has been reported to associate with SGs under certain conditions [31, 66]. For example, Colombrita et al. [66] generated sodium arsenite-induced TDP-43/TIAR immunopositive inclusions in a TDP43 over-expressing system. Other studies have previously reported findings on TDP-43 in non-TIAR positive structures in agreement with our findings [35, 55, 67], including generation of the same pattern of sorbitol-induced punctate TDP-43 accumulation in non-TIAR positive SGs [67]. It is of interest that hyperosmolar stress has also been shown to cause mislocalisation of FUS [68]. The relevance of this to sporadic MND is the implication that the development of TDP-43 aggregates in patients and the precise location and co-localisation with other markers could reflect differences in underlying cellular stresses. It is possible that, in patients with sporadic disease, different cellular stresses, for example ER stress and oxidative stress, lead to TDP-43 aggregation through distinct pathways and this may account for the recent finding of different TDP-43 inclusion morphologies in MND and frontotemporal dementia, namely the rounded and circumferential morphologies [58]. Furthermore, the differences in underlying stress may account the multiplicity of signalling pathways and TDP-43 kinases implicated in neurodegeneration, ranging from CK1 to cyclin-dependent kinase and glycogen synthase kinase 3 [22, 23, 25, 69, 70].

Biochemical methods were used to understand the relationship between accumulation of TDP-43, phosphorylation and potential aggregation. Immunoblotting data showed that tunicamycin treatment did not have any significant impact on levels of total TDP-43 or phospho-TDP-43 in the RIPA-soluble fraction (Fig. 4) and the very low pTDP-43 immunoreactivity agrees with the findings of others [71]. However, it would be expected that any aggregated species would be present in the RIPA-insoluble, urea-soluble fraction. Indeed this proved to be the case, with substantial increases in multiple phosphorylated TDP-43 species. The specificity of the higher molecular weight species was demonstrated by the inclusion of the reducing agent DTT in the sample buffer in a subset of samples. This caused a decrease in immunoreactivity of the higher molecular weight phospho-TDP-43 species, confirming the findings of Cohen et al. [54], who found higher order TDP-43 species to be dependent on disulphide bonds. The tunicamycin-induced increase in phospho-TDP-43 was lowered by the CK1 inhibitors, D4476 and PF670462. The ability of kinase inhibition to block ER-induced phosphorylation of TDP-43 and concurrently, TDP-43 cytoplasmic accumulation, suggests that stress-induced TDP-43 phosphorylation is an early step in sporadic MND, followed by accumulation of TDP-43 in cytoplasmic inclusions in the RIPA-insoluble fraction. This is in line with previous findings, which showed the ability of D4476 to reduce the ethacrynic acid-induced phosphorylation of TDP-43 [72].

Furthermore, in this study, TDP-43 phosphorylation and accumulation were not accompanied by any increase in cellular LDH release or reduction in the cells’ ability to metabolise the MTS tetrazolium dye (at 0.1 µM tunicamycin). This finding suggests that our system may be modelling the early stages of sporadic disease, showing that inclusions of TDP-43 are not initially concurrent with cell death or metabolic disturbance. This is consistent with the notion of TDP-43 accumulating as part of a protective response to stress, analogous to the role of stress granules. However, if the concentration of tunicamycin is raised to 0.5 µM or above, then the cells exhibit substantial deficits in their ability to metabolise the MTS tetrazolium dye. Notably, cells still do not exhibit signs of frank cell death (i.e. membrane permeabilisation), even at the highest concentrations of tunicamycin. Co-incubation of cells with a CK1 inhibitor shows this to have a beneficial effect at the earliest stages, i.e. blocking TDP-43 phosphorylation and cytosolic accumulation. However, once metabolic disturbances occur at higher concentrations of tunicamycin, kinase inhibition has no effect. This paradigm has been recapitulated in a genetic model of MND, over-expressing mutant TDP-43, where TDP-43 aggregation preceded any cell death [73].

Recent work has shown a correlation between phospho-TDP-43 and CK1 levels in sporadic MND patients and that CK1ε could act as a TDP-43 kinase in human motor neurons [21]. Motor neurones derived from C9ORF72 hexanucleotide expansion carriers display a similar phenotype to the NSC-34 cells in this study, in terms of increased expression of Grp78 [74]. Also, circulating lymphocytes from individuals with sporadic MND show no change in total TDP-43, but CK1-dependent increases in phospho-TDP-43, as seen in our study. [75]. Taken together these findings suggest that our model replicates some of the pathological correlates of sporadic MND in humans.

The data shown here give insight into the chronology of events in a sporadic MND model. On encountering ER stress, cells up regulate chaperone expression e.g. Grp78. Signalling downstream of cellular stress also results in activation of kinases, such as CK1, which lead to phosphorylation of TDP-43 and accumulation in the insoluble fraction. At this point, the cells do not display any metabolic deficiencies and the changes in TDP-43 modification and localization can be mitigated by small molecule CK1 inhibitors. However, at higher levels of stress, reduced ability to metabolise MTS tetrazolium is observed and at this stage, CK1 inhibitors have no effect. This underscores the importance of early diagnosis of MND as it suggests that the earliest pathological changes may be reversed by small molecules, but that this approach may be less effective once there is metabolic disturbance in the motor neurons.

## Electronic supplementary material

Below is the link to the electronic supplementary material.

Supplementary material 1 (PDF 1040 kb) **Fig. S1** Cellular response to endoplasmic reticulum stress. NSC-34 cells were cultured as described, differentiated for seven days and treated with 0.1 µM tunicamycin (24 h). Cells were lysed and proteins analyzed by western blotting. **(a)** Primary antibodies were against Grp78, TDP-43 and β-actin, with **(b)** densitometric analysis of normalized TDP-43 and Grp78 performed. **(c)** Cell viability was assessed using the CellTiter-Glo assay. Each point represents a biological replicate (n = 3) with error bars as SD. Horizontal lines (black) indicate mean and SD Statistical significance was assessed using Student’s t test with Holm-Sidak correction. **Fig. S2** Validation of phospho-TDP-43 species. **(a)** NSC-34 cells were lysed in minimal lysis buffer and incubated with λ phosphatase, followed by SDS-PAGE and immunoblotting, probing for pTDP-43. **(b)** RIPA-insoluble (tunicamycin-treated) samples were separated by SDS-PAGE (± 1 mM DTT in the sample buffer), followed by immunoblotting for pTDP-43. * = high molecular weight pTDP-43 and # = monomeric pTDP-43

## Abbreviations

Cdk: Cyclin-dependent kinase
CK: Casein kinase
CHOP: C/EBP homologous protein
ERK: Extracellular signal regulated kinase
FUS: Fused in sarcoma
G3BP1: Ras GTPase-activating protein-binding protein 1
GSK-3β: Glycogen synthase kinase-3 beta
JNK: c-Jun N-terminal kinase
MND: Motor neuron disease
PDI: Protein disulphide isomerase
RRID: Research resource identifier
SG: Stress granule
(p) TDP-43: (phosphorylated) TAR DNA binding protein 43
TIAR: T-intracellular antigen-1 cytotoxic granule-associated RNA binding protein-like 1
