# Supplementary material for: Endoplasmic Reticulum Stress Signalling Induces Casein Kinase 1-Dependent Formation of Cytosolic TDP-43 Inclusions in Motor Neuron-Like Cells
Source: Neurochem Res. 2019 Jul 6;45(6):1354–64. doi: 10.1007/s11064-019-02832-2 (PMC7260270; doi:10.1007/s11064-019-02832-2)
Supplement: Supplementary file 1 — Supplementary material 1 (PDF 1040 kb) Fig. S1 Cellular response to endoplasmic reticulum stress. NSC-34 cells were cultured as described, differentiated for seven days and treated with 0.1 µM tunicamycin (24 h). Cells were lysed and proteins analyzed by western blotting. (a) Primary antibodies were against Grp78, TDP-43 and β-actin, with (b) densitometric analysis of normalized TDP-43 and Grp78 performed. (c) Cell viability was assessed using the CellTiter-Glo assay. Each point represents a biological replicate (n = 3) with error bars as SD. Horizontal lines (black) indicate mean and SD Statistical significance was assessed using Student’s t test with Holm-Sidak correction. Fig. S2 Validation of phospho-TDP-43 species. (a) NSC-34 cells were lysed in minimal lysis buffer and incubated with λ phosphatase, followed by SDS-PAGE and immunoblotting, probing for pTDP-43. (b) RIPA-insoluble (tunicamycin-treated) samples were separated by SDS-PAGE (± 1 mM DTT in the sample buffer), followed by immunoblotting for pTDP-43. * = high molecular weight pTDP-43 and # = monomeric pTDP-43 [file 11064_2019_2832_MOESM1_ESM.pdf]

Endoplasmic reticulum stress signalling induces casein kinase 1-dependent formation of cytosolic TDP-43 inclusions in motor neuron-like cells

David A. Hicks[1,2]*, Laura L. Cross[1], Ritchie Williamson[1], and Marcus Rattray[1]*

[1] *School of Pharmacy and Medical Sciences, Faculty of Life Sciences, University of Bradford, Richmond Road, Bradford, BD7 1DP, United Kingdom*

[2] *Current address: Division of Neuroscience & Experimental Psychology, School of Biological Sciences, Faculty of Biology, Medicine and Health, University of Manchester, Oxford Road, Manchester, M13 9PT*

*To whom correspondence and reprint requests should be addressed:

David A. Hicks, Faculty of Biology, Medicine and Health, AV Hill Building, University of Manchester; david.hicks-2@manchester.ac.uk; Tel +44 (0)161 3060502; ORCiD: 0000-0001-6045-1063

Marcus Rattray, School of Pharmacy and Medical Sciences, Faculty of Life Sciences, University of Bradford; m.rattray@bradford.ac.uk; Tel +44 (0)1274 234675

Running title: Induction of TDP-43 cytoplasmic accumulation

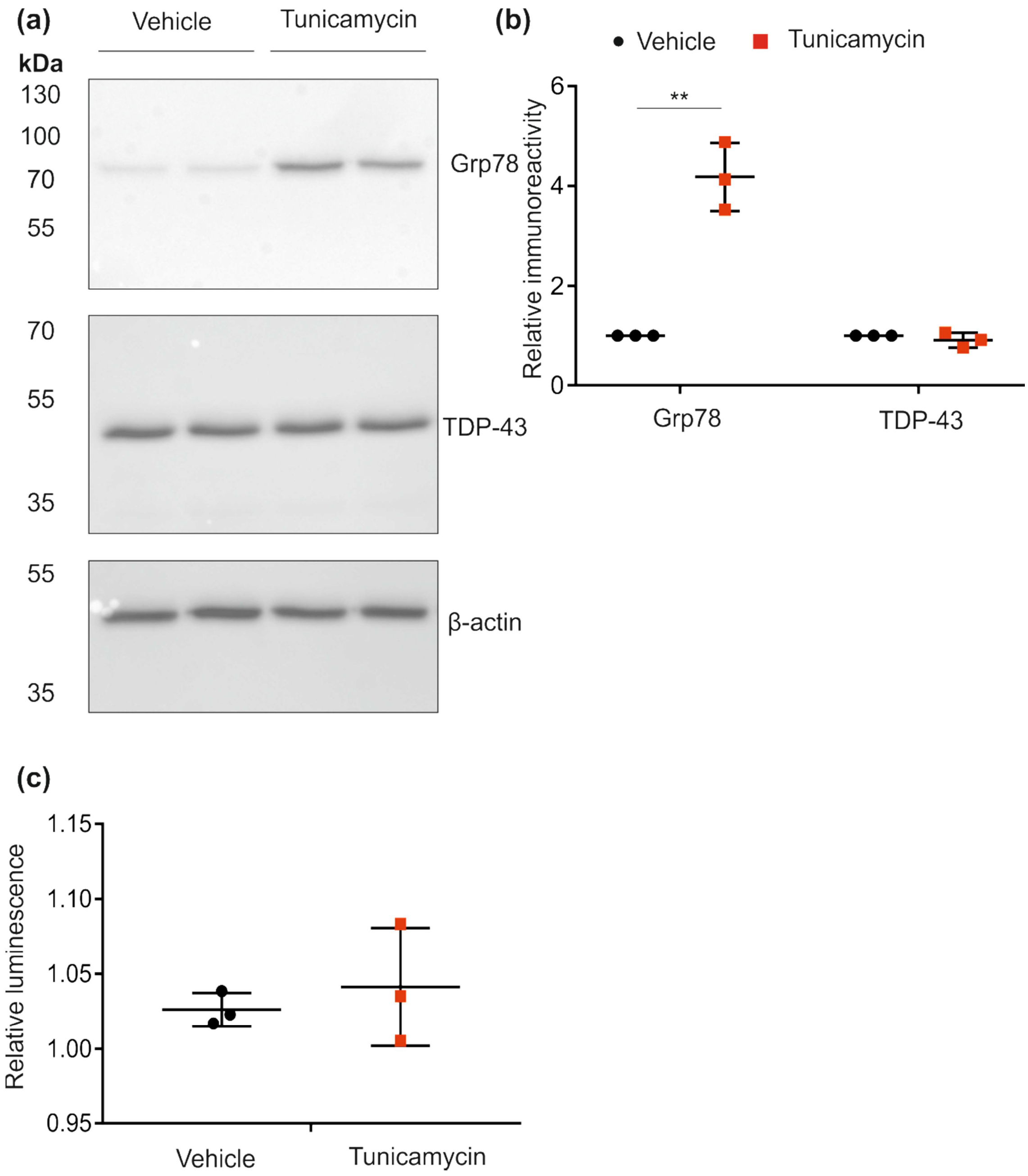
(a)
Vehicle
Tunicamycin
kDa
130
100
70
55
Grp78
70
55
TDP-43
35
55
β-actin
35
(b)
Vehicle
Tunicamycin
Relative immunoreactivity
6
4
2
0
**
Grp78
TDP-43
(c)
Relative luminescence
1.15
1.10
1.05
1.00
0.95
Vehicle
Tunicamycin

Figure S1

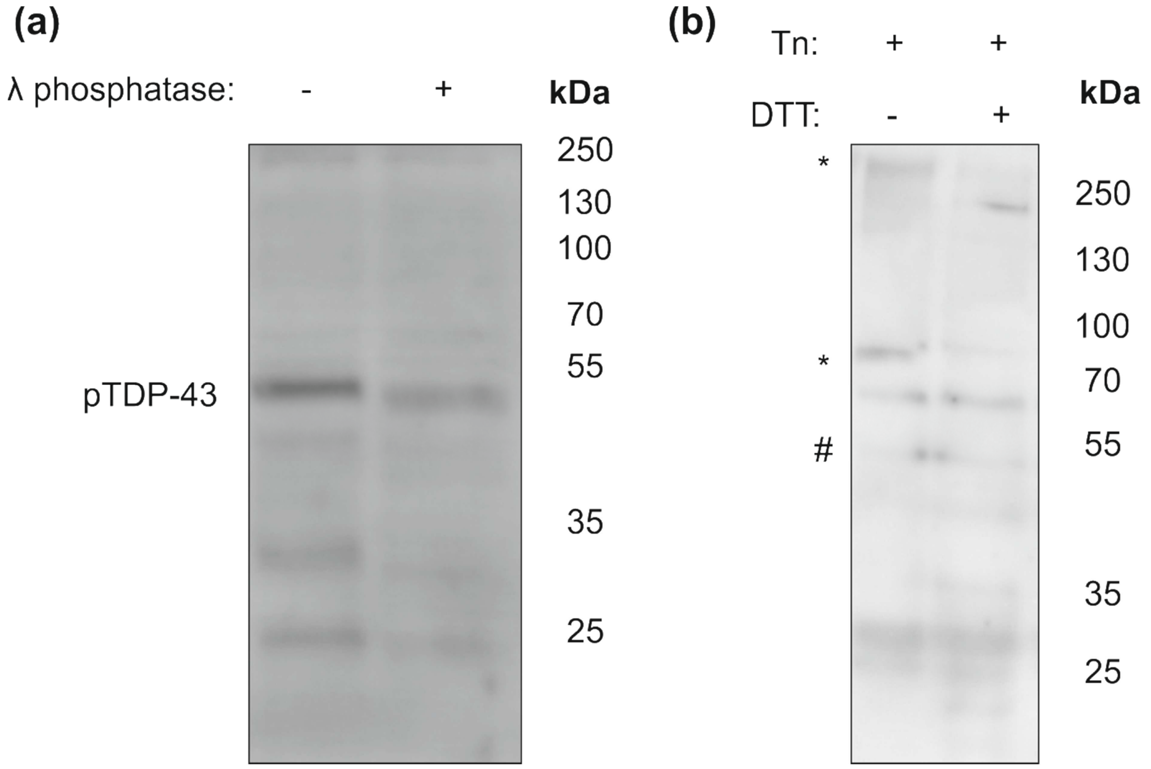

(a)
λ phosphatase:
-
+
kDa
250
130
100
70
55
pTDP-43
35
25
(b)
Tn:
+
+
DTT:
-
+
kDa
*
250
130
100
*
70
#
55
35
25

Figure S2
